# Supplementary material for: Effect of Single Nucleotide Polymorphisms in the Vitamin D Metabolic Pathway on Susceptibility to Non-Small-Cell Lung Cancer
Source: Nutrients. 2022 Nov 4;14(21):4668. doi: 10.3390/nu14214668 (PMC9659229; doi:10.3390/nu14214668)
Supplement: Supplementary file 1 [file nutrients-14-04668-s001.zip › Table S1.pdf]

Table S1. Hardy-Weinberg Equilibrium.

Chr	SNP	Sample	Minor Allele	Major Allele	Genotype counts	Observed heterozygosity	Expected heterozygosity	p-value
4	rs7041	ALL	T	G	120/304/183	0.5008	0.4946	0.8056
4	rs7041	AFF	T	G	43/92/68	0.4532	0.4924	0.2565
4	rs7041	UNAFF	T	G	77/212/115	0.5248	0.4956	0.2697
11	rs10741657	ALL	A	G	96/269/234	0.4491	0.4735	0.2267
11	rs10741657	AFF	A	G	26/97/78	0.4826	0.4665	0.7621
11	rs10741657	UNAFF	A	G	70/172/156	0.4322	0.4767	0.07342
12	rs731236	ALL	C	T	94/298/212	0.4934	0.4809	0.5543
12	rs731236	AFF	C	T	21/110/72	0.5419	0.4684	0.03542
12	rs731236	UNAFF	C	T	73/188/140	0.4688	0.486	0.4734
12	rs7975232	ALL	C	A	133/301/173	0.4959	0.4978	0.9351
12	rs7975232	AFF	C	A	54/98/52	0.4804	0.5	0.5772
12	rs7975232	UNAFF	C	A	79/203/121	0.5037	0.4946	0.7628
12	rs1544410	ALL	A	G	122/289/197	0.4753	0.4924	0.4102
12	rs1544410	AFF	A	G	25/108/71	0.5294	0.4746	0.1393
12	rs1544410	UNAFF	A	G	97/181/126	0.448	0.4974	0.04586
12	rs2228570	ALL	T	C	79/275/254	0.4523	0.4586	0.7242
12	rs2228570	AFF	T	C	24/90/89	0.4433	0.4487	0.8761
12	rs2228570	UNAFF	T	C	55/185/165	0.4568	0.4631	0.83
12	rs11568820	ALL	A	G	34/231/335	0.385	0.3742	0.5143
12	rs11568820	AFF	A	G	11/73/120	0.3578	0.3573	1
12	rs11568820	UNAFF	A	G	23/158/215	0.399	0.3825	0.4326
12	rs4646536	ALL	G	A	47/219/343	0.3596	0.3819	0.1671
12	rs4646536	AFF	G	A	15/70/118	0.3448	0.3713	0.3434
12	rs4646536	UNAFF	G	A	32/149/225	0.367	0.387	0.3052
12	rs3782130	ALL	C	G	43/218/341	0.3621	0.3775	0.3311
12	rs3782130	AFF	C	G	13/69/120	0.3416	0.3597	0.4399
12	rs3782130	UNAFF	C	G	30/149/221	0.3725	0.386	0.5168
12	rs10877012	ALL	T	G	45/221/338	0.3659	0.3823	0.2881
12	rs10877012	AFF	T	G	13/70/120	0.3448	0.3611	0.5592
12	rs10877012	UNAFF	T	G	32/151/218	0.3766	0.3924	0.4448
12	rs703842	ALL	C	T	42/215/344	0.3577	0.3737	0.3254
12	rs703842	AFF	C	T	16/71/116	0.3498	0.3787	0.2694
12	rs703842	UNAFF	C	T	26/144/228	0.3618	0.3712	0.591
20	rs4809957	ALL	G	A	26/213/359	0.3562	0.345	0.4779
20	rs4809957	AFF	G	A	9/68/126	0.335	0.3339	1
20	rs4809957	UNAFF	G	A	17/145/233	0.3671	0.3505	0.3915
20	rs6068816	ALL	T	C	10/119/477	0.1964	0.2031	0.4214
20	rs6068816	AFF	T	C	7/44/152	0.2167	0.2449	0.1433
20	rs6068816	UNAFF	T	C	3/75/325	0.1861	0.1808	0.7832

Chr: Chromosome; ALL: All population; AFF: Case group; UNAFF: Control group; Shade means the value is significant
