# Supplementary material for: Effect of Single Nucleotide Polymorphisms in the Vitamin D Metabolic Pathway on Susceptibility to Non-Small-Cell Lung Cancer
Source: Nutrients. 2022 Nov 4;14(21):4668. doi: 10.3390/nu14214668 (PMC9659229; doi:10.3390/nu14214668)
Supplement: Supplementary file 1 [file nutrients-14-04668-s001.zip › Table S3.pdf]

Table S3. Minor allele frequencies of SNPs.

Chr	SNP	Minor Allele	Major Alelle	MAF
4	rs7041	T	G	0.4481
11	rs10741657	A	G	0.3848
12	rs731236	C	T	0.4023
12	rs7975232	C	A	0.4671
12	rs1544410	A	G	0.4383
12	rs2228570	T	C	0.3561
12	rs11568820	A	G	0.2492
12	rs4646536	G	A	0.257
12	rs3782130	C	G	0.2525
12	rs10877012	T	G	0.2575
12	rs703842	C	T	0.2488
20	rs4809957	G	A	0.2216
20	rs6068816	T	C	0.1147
Chr: Chromosome; MAF: Minor allele frequency				
