# Supplementary material for: Effect of Single Nucleotide Polymorphisms in the Vitamin D Metabolic Pathway on Susceptibility to Non-Small-Cell Lung Cancer
Source: Nutrients. 2022 Nov 4;14(21):4668. doi: 10.3390/nu14214668 (PMC9659229; doi:10.3390/nu14214668)
Supplement: Supplementary file 1 [file nutrients-14-04668-s001.zip › Table S4.pdf]

Table S4. Haplotype frequency estimates.

	rs1544410	rs7975232	rs731236	rs4646536	rs703842	rs3782130	rs10877012	Total	Case group	Control group	Cumulative frequency
1	G	C	T	A	T	G	G	0.3294	0.4048	0.2895	0.3294
2	A	A	C	A	T	G	G	0.2379	0.2167	0.2455	0.5672
3	A	A	C	G	C	C	T	0.103	0.1386	0.0889	0.6702
4	G	A	T	A	T	G	G	0.0799	0.1006	0.0724	0.7501
5	G	C	T	G	C	C	T	0.0667	0.0757	0.0606	0.8168
6	A	C	T	A	T	G	G	0.0231	NA	0.0356	0.8399
7	G	A	T	G	C	C	T	0.0208	0.013	0.0217	0.8607
8	A	A	T	A	T	G	G	0.0145	0.0124	0.0155	0.8752
9	G	A	C	A	T	G	G	0.013	0.0025	0.0188	0.8883
10	G	C	T	G	T	C	T	0.0106	NA	0.0168	0.8989
11	A	A	C	G	T	C	T	0.0083	0.0025	0.0116	0.9072
12	A	A	T	G	C	C	T	0.0079	NA	0.0125	0.915
13	G	C	T	A	C	G	G	0.0075	0.0093	0.0072	0.9225
14	A	A	C	A	C	G	G	0.0071	0.0029	0.009	0.9297
15	G	C	T	A	T	C	G	0.0051	NA	0.0081	0.9348
16	A	C	T	G	C	C	T	0.0047	NA	0.007	0.9395
17	A	A	C	G	C	C	G	0.0042	NA	0.0058	0.9437
18	A	A	C	G	T	G	T	0.0038	NA	0.0057	0.9475
19	G	C	T	G	C	G	G	0.0037	0.0041	0.0037	0.9511
20	G	C	T	A	T	G	T	0.0034	NA	0.0052	0.9546
21	A	A	C	A	T	G	T	0.0033	NA	0.0051	0.9579
22	A	A	C	A	C	C	T	0.0033	NA	0.005	0.9612
23	G	A	T	G	T	C	T	0.0031	0.0025	0.0033	0.9644
24	A	C	C	A	T	G	G	0.0029	0.0036	0.0026	0.9672
25	G	C	T	G	C	G	T	0.0028	NA	0.0049	0.97
26	A	A	C	A	T	C	G	0.0026	NA	0.0037	0.9726
27	G	A	C	G	C	C	T	0.0024	NA	0.0041	0.975
28	G	A	C	G	T	C	T	0.0022	NA	0.0029	0.9772
29	A	C	C	G	C	C	T	0.0021	0.0048	4e-04	0.9793
30	G	A	T	A	T	G	T	0.0019	NA	0.0029	0.9811
31	G	C	T	G	C	C	G	0.0018	NA	0.0032	0.983
32	G	A	T	A	C	G	G	0.0017	2e-04	0.0023	0.9847
33	A	A	C	G	T	C	G	0.0017	NA	0.0025	0.9863
34	A	A	T	A	T	G	T	0.0016	NA	0.0025	0.9879
35	G	A	T	G	C	C	G	0.0015	NA	0.0022	0.9894
36	A	A	C	G	C	G	G	0.0012	0.0032	NA	0.9906
37	G	C	C	A	T	G	G	0.0011	NA	0.0016	0.9917
38	G	A	C	G	C	G	T	9e-04	NA	0.0014	0.9926
39	A	C	C	G	C	G	G	9e-04	0.0025	NA	0.9934
40	A	A	C	G	T	G	G	9e-04	NA	0.0013	0.9943
41	A	A	T	A	T	C	G	8e-04	NA	0.0012	0.9951
42	A	A	T	G	C	G	G	8e-04	NA	0.0012	0.9959
43	G	A	T	A	C	G	T	8e-04	NA	0.0011	0.9967
44	G	C	C	G	C	C	T	7e-04	NA	0.0011	0.9975
45	G	A	C	A	T	C	G	7e-04	NA	8e-04	0.9981
46	A	C	T	A	C	G	G	7e-04	NA	9e-04	0.9988
47	A	A	C	G	C	G	T	5e-04	NA	NA	0.9993
48	A	A	T	G	T	C	T	4e-04	NA	0	0.9996
49	A	A	T	A	C	G	G	3e-04	NA	4e-04	0.9999
50	A	C	T	G	T	C	T	1e-04	NA	NA	1
51	G	C	T	G	T	C	G	0	NA	0	1
52	G	C	T	A	C	C	T	0	NA	0	1

* Rare haplotypes; NA: not available
