# Supplementary material for: Effect of Single Nucleotide Polymorphisms in the Vitamin D Metabolic Pathway on Susceptibility to Non-Small-Cell Lung Cancer
Source: Nutrients. 2022 Nov 4;14(21):4668. doi: 10.3390/nu14214668 (PMC9659229; doi:10.3390/nu14214668)
Supplement: Supplementary file 1 [file nutrients-14-04668-s001.zip › Table S5.pdf]

Table S5. Polymorphisms and association with risk of NSCLC.

SNP	Minor Allele	Mayor Allele	Model	Cases	Controls	χ²	p-value χ²	p-value Fisher	Adjusted p-value*
rs1544410	A	G	Genotypic	25/108/71	97/181/126	11.77	0.00278	0.002042	0.0361
			Additive	158/250	375/433	6.286	0.01217	0.01217	0.1582
			Allelic	158/250	375/433	6.504	0.01076	0.01206	0.1399
			Dominant	133/71	278/126	0.8091	0.3684	0.4089	1
			Recessive	25/179	97/307	11.68	0.000632	0.0005676	0.0082
rs11568820	A	G	Genotypic	11/73/120	23/158/215	1.128	0.5689	0.563	1
			Additive	95/313	204/588	0.9067	0.341	0.341	1
			Allelic	95/313	204/588	0.8805	0.3481	0.3605	1
			Dominant	84/120	181/215	1.121	0.2898	0.299	1
			Recessive	11/193	23/373	0.04357	0.8347	1	1
rs2228570	T	C	Genotypic	24/90/89	55/185/165	0.6869	0.7093	0.7302	1
			Additive	138/268	295/515	0.6869	0.4072	0.4072	1
			Allelic	138/268	295/515	0.6963	0.404	0.41	1
			Dominant	114/89	240/165	0.5348	0.4646	0.4861	1
			Recessive	24/179	55/350	0.3695	0.5433	0.6097	1
rs7975232	C	A	Genotypic	54/98/52	79/203/121	4.041	0.1326	0.1389	1
			Additive	206/202	361/445	3.523	0.06051	0.06051	0.7866
			Allelic	206/202	361/445	3.537	0.06001	0.06768	0.7801
			Dominant	152/52	282/121	1.367	0.2424	0.2547	1
			Recessive	54/150	79/324	3.733	0.05333	0.06145	0.6933
rs731236	C	T	Genotypic	21/110/72	73/188/140	6.819	0.03306	0.02979	0.4297
			Additive	152/254	334/468	2.037	0.1535	0.1535	1
			Allelic	152/254	334/468	1.984	0.1589	0.1719	1
			Dominant	131/72	261/140	0.01824	0.8926	0.9282	1
			Recessive	21/182	73/328	6.336	0.01183	0.01242	0.1537
rs7041	T	G	Genotypic	43/92/68	77/212/115	2.824	0.2437	0.2445	1
			Additive	178/228	366/442	0.2342	0.6285	0.6285	1
			Allelic	178/228	366/442	0.2312	0.6306	0.6686	1
			Dominant	135/68	289/115	1.625	0.2024	0.2231	1
			Recessive	43/92/68	77/212/115	2.824	0.2437	0.2445	1
rs10741657	A	G	Genotypic	43/160	77/327	0.3839	0.5355	0.5893	1
			Additive	26/97/78	70/172/156	2.565	0.2773	0.281	1
			Allelic	149/253	312/484	0.4874	0.4851	0.4851	1
			Dominant	149/253	312/484	0.5125	0.474	0.4896	1
			Recessive	123/78	242/156	0.008534	0.9264	1	1
rs4646536	G	A	Genotypic	15/70/118	32/149/225	0.4039	0.8171	0.8216	1
			Additive	100/306	213/599	0.3433	0.5579	0.5579	1
			Allelic	100/306	213/599	0.3633	0.5467	0.5782	1
			Dominant	85/118	181/225	0.4038	0.5251	0.5449	1
			Recessive	15/188	32/374	0.04611	0.83	0.8736	1
rs3782130	C	G	Genotypic	13/69/120	30/149/221	0.9764	0.6137	0.6267	1
			Additive	95/309	209/591	0.9311	0.3346	0.3346	1
			Allelic	95/309	209/591	0.969	0.3249	0.3611	1
			Dominant	82/120	179/221	0.944	0.3313	0.3395	1
			Recessive	13/189	30/370	0.2292	0.6321	0.7383	1
rs10877012	T	G	Genotypic	13/70/120	32/151/218	1.363	0.5057	0.5184	1
			Additive	96/310	215/587	1.352	0.2449	0.2449	1
			Allelic	96/310	215/587	1.41	0.235	0.265	1
			Dominant	83/120	183/218	1.233	0.2667	0.2979	1
			Recessive	13/190	32/369	0.4855	0.4859	0.5174	1
rs703842	C	T	Genotypic	16/71/116	26/144/228	0.4052	0.8166	0.8139	1
			Additive	103/303	196/600	0.07685	0.7816	0.7816	1
			Allelic	103/303	196/600	0.08014	0.7771	0.7782	1
			Dominant	87/116	170/228	0.001132	0.9732	1	1
			Recessive	16/187	26/372	0.3764	0.5395	0.6121	1
rs4809957	G	A	Genotypic	9/68/126	17/145/233	0.6055	0.7388	0.7373	1
			Additive	86/320	179/611	0.3501	0.554	0.554	1

			Allelic	86/320	179/611	0.3387	0.5606	0.6069	1
			Dominant	77/126	162/233	0.5307	0.4663	0.4818	1
			Recessive	9/194	17/378	0.005424	0.9413	1	1
rs6068816	T	C	Genotypic	7/44/152	3/75/325	NA	NA	0.02918	NA
			Additive	58/348	81/725	4.619	0.03161	0.03161	0.4109
			Allelic	58/348	81/725	4.772	0.02893	0.03532	0.3761
			Dominant	51/152	78/325	NA	NA	0.1148	NA
			Recessive	7/196	3/400	NA	NA	0.01937	NA
Chr: chromosome; NA: not applicable; * p-value for Bonferroni correction; Shade means the value is significant									
